# Metal-Semiconductor-Metal GeSn Photodetectors on Silicon for Short-Wave Infrared Applications

**DOI:** 10.3390/mi11090795

**Published:** 2020-08-21

**Authors:** Soumava Ghosh, Kuan-Chih Lin, Cheng-Hsun Tsai, Harshvardhan Kumar, Qimiao Chen, Lin Zhang, Bongkwon Son, Chuan Seng Tan, Munho Kim, Bratati Mukhopadhyay, Guo-En Chang

**Affiliations:** 1 Department of Mechanical Engineering, and Advanced Institute of Manufacturing with High-Tech Innovations (AIM-HI), National Chung Cheng University, Chiyai County 62102, Taiwan; ghoshsoumava2@gmail.com (S.G.); harshvardhan@nitdelhi.ac.in (H.K.); 2 Graduate Institute of Opto-Mechatronics, National Chung Cheng University, Chiyai County 62102, Taiwan; xavierlin0105@gmail.com (K.-C.L.); a0918663250@gmail.com (C.-H.T.); 3 Institute of Radio Physics and Electronics, University of Calcutta, Kolkata 700009, India; bratmuk@yahoo.co.in; 4 School of Electrical and Electronic Engineering, Nanyang Technological University, Singapore 639798, Singapore; chenqm@ntu.edu.sg (Q.C.); zhang.lin@ntu.edu.sg (L.Z.); BONGKWON001@e.ntu.edu.sg (B.S.); TanCS@ntu.edu.sg (C.S.T.); munho.kim@ntu.edu.sg (M.K.)

**Keywords:** photodetectors, GeSn alloys, silicon photonics, photonic integrated circuits

## Abstract

Metal-semiconductor-metal photodetectors (MSM PDs) are effective for monolithic integration with other optical components of the photonic circuits because of the planar fabrication technique. In this article, we present the design, growth, and characterization of GeSn MSM PDs that are suitable for photonic integrated circuits. The introduction of 4% Sn in the GeSn active region also reduces the direct bandgap and shows a redshift in the optical responsivity spectra, which can extend up to 1800 nm wavelength, which means it can cover the entire telecommunication bands. The spectral responsivity increases with an increase in bias voltage caused by the high electric field, which enhances the carrier generation rate and the carrier collection efficiency. Therefore, the GeSn MSM PDs can be a suitable device for a wide range of short-wave infrared (SWIR) applications.

## 1. Introduction

The monolithic integration of photonic components and devices on a silicon (Si) platform to create electronic–photonic integrated circuits (EPICs) has attracted immense research interest over recent decades [1,2,3,4,5]. The key driving force is the bottleneck in the data transport rate due to the limited data rates of~10 GB/s of the current copper interconnects. Nowadays, optical and photonic interconnects operating in the fiber-optic low-loss windows (1264–1675 nm) of silica fibers have been proposed to replace metal interconnects to increase the data transport rate and reduce thermal problems. Among the various active photonic components, photodetectors (PDs) are a crucial building block. Although III-V semiconductor compounds are mostly used for high-speed photodetection, these compounds are not compatible with the Si complementary metal-oxide-semiconductor (CMOS) IC technology [6]. 

Alternatively, the compatibility with Si-based CMOS processing technology, monolithic integration on the same Si chip [7], and low fabrication cost make group-IV materials very attractive to develop CMOS-compatible photonic devices. However, the realization of those devices for the most important fiber-optical communication window (1310 and 1550 nm) by Si is not possible due to its bandgap of 1.12 eV [8], resulting in a cutoff wavelength of ~1100 nm. This problem can be partially circumvented by using Ge, as its direct bandgap (0.8 eV) supports 1310 nm at room temperature. However, beyond 1500 nm wavelength, the responsivity of Ge falls drastically [9]. Therefore, the entire telecommunication bands cannot be covered by Ge based photodetectors (PDs). 

Over recent decades, the growth of high-quality Ge_1-x_Sn_x_ thin film by chemical vapor deposition (CVD) and molecular beam epitaxy (MBE) [10,11,12] on Si substrate via a suitable buffer has opened new avenues for group-IV photonics. The incorporation of Sn in Ge not only modifies the electronic band structure by shrinking the direct bandgap and hence red-shifting the absorption edge [13], but also beyond 8% Sn concentration the GeSn alloy acts as a direct bandgap semiconductor [14]. This noteworthy feature of GeSn has encouraged researchers to develop different types of optoelectronic devices such as Light Emitting Diodes (LEDs) [15,16,17], LASERs [18,19,20], Transistor LASERs [21,22], *p-i-n* PDs [23,24,25], quantum well infrared photodetectors (QWIPs) [26,27,28], metal-semiconductor-metal photodetectors (MSM PDs) [29,30,31], waveguide PDs [32], and heterojunction bipolar phototransistors (HPTs) [33,34,35,36,37,38,39,40]. 

MSM PDs are an alternative choice of *p-i-n* PD, consisting of back-to-back Schottky diodes. As MSM PDs do not require any doping, the effect of parasitic capacitance cannot degrade the performance [41]. Only transit-time limited delay presents; therefore, the operation speed is higher than normal *p-i-n* PDs. The simple planar fabrication of MSM PDs is suitable for monolithic integration with other components of photonic circuits [42,43]. Yasar et al. [29] and Mahmodi et al. [30] reported amorphous (8% Sn content) and crystalline GeSn MSM PD on Si substrates, respectively, but they only focused on the measurement of dark and photocurrent. Recently, the responsivity of Ge_1-x_Sn_x_ thin-film based MSM PD on Si has been reported [31]; however, in their work, they only demonstrated up to 1000 nm wavelength which cannot cover the modern telecommunication window (1550 nm). 

In this work, we demonstrate the GeSn MSM PD on the Si platform for efficient photodetection in the entire telecommunication bands. We show the material growth and electrical and optical characterization results of the fabricated GeSn MSM PD with 4% Sn content. Furthermore, we also measured the spectral responsivity for different bias voltages and analyzed the strain electronic band structure and absorption to study the enhanced photodetection. 

## 2. Materials and Methods

### 2.1. Device Design 

**Figure 1:**
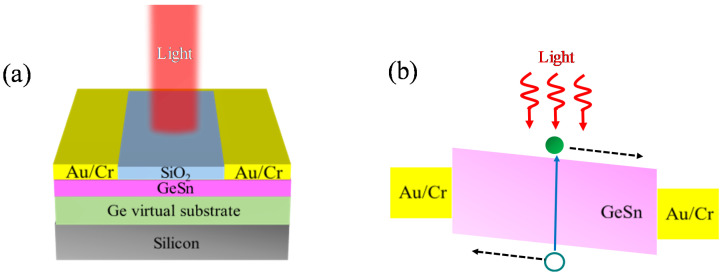
(**a**) 3D Schematic diagram of our designed surface-illuminated GeSn metal-semiconductor-metal photodetectors (MSM PDs) on Ge buffered Si substrates. (**b**) Schematic band diagram of the GeSn MSM PD.

Figure 1a exhibits the 3D schematic diagram of the designed surface-illuminated GeSn MSM PD. The layer structure consisted of a GeSn active layer as the absorption layer grown on the Si substrate via Ge virtual substrate (VS). The presence of GeSn active region helped to enhance the absorption capacity with respect to the pure Ge, due to the smaller direct bandgap and the larger absorption coefficient. The GeSn layer was passivated by the SiO_2_ layer. Two metal pads were deposited on the top surface of the GeSn active layer. A schematic band diagram is shown in Figure 1b.

As light is normally incident on the device and absorbed by the GeSn active layer, electrons and holes could be generated in the GeSn active layer. In the presence of the bias voltage, the potential difference between positive and negative electrical contacts created a band bending. Under the illuminated conditions, the electron-hole pairs were generated in the active region and then swept out to the electrodes in the presence of the induced electric field. The holes and electrons accumulated in the negative and positive metal contacts, respectively, resulting in the flow of the photocurrent. The materials and dimensions of the different regions are listed in Table 1.

### 2.2. Material Growth and Characterization

The GeSn sample was grown on a 150 mm Si (001) substrate in industry compatible reduced pressure-chemical vapor deposition (RP-CVD) chamber from ASM by using Ge_2_H_6_ and SnCl_4_ as the precursors. Prior to the deposition of the GeSn layer, a high-quality Ge VS with a thickness of ~900 nm was deposited on the Si substrate by using Ge_2_H_6_ as the precursor at 400 °C followed by annealing at 850 °C for 30 min. After that, the temperature in the chamber was decreased to 325 °C, and a 180 nm thick of GeSn film was deposited on Ge VS as the active layer. The Sn concentration and strain of the GeSn layer were characterized at room temperature by X-ray diffraction reciprocal space mapping (XRDRSM) using a PANalytical X’Pert diffractometer. The microstructure of the GeSn sample was investigated by cross-sectional transmission electron microscopy (XTEM) in dark field mode (FEI Tecnai G2 F20, FEI, Waltham, MA, USA). A Pt layer was deposited on the surface of the GeSn sample to increase the conductivity for XTEM experiments.

### 2.3. MSM PD Fabrication

The surface-illuminated GeSn MSM PDs were fabricated using a standard CMOS-compatible process. A square mesa with a width of 1 mm was created by standard optical lithography followed by reactive ion etching (RIE) techniques. Next, a 180 nm thick SiO_2_ passivation layer was deposited using plasma-enhanced chemical vapor deposition (PECVD), which acts as an electric isolator between positive and negative electrodes and an anti-reflection (AR) layer to enhance the optical responsivity. After that, contact windows were incorporated using optical lithography and wet etching methods with buffered oxide etch (BOE) solution to expose the semiconductor surfaces for making the electrical contacts. Finally, 200/20 nm thick Au/Cr metal pads were deposited using an e-beam evaporator and patterned rectangular-shaped using a lift-off process.

### 2.4. Electrical and Optical Measurements

A Keithley 2400 SourceMeter was used to characterize the electrical properties of the fabricated GeSn MSM PDs at room temperature under dark and illuminated conditions. To characterize the spectral property of the GeSn MSM PDs, Fourier-transform infrared spectroscopy (FTIR) was used. The emitted light from the FTIR was incident normally on the devices. A Keithley 2400 SourceMeter and a 50 Ω load resistance in series were used to bias the GeSn MSM PDs. The voltage drop across the load resistance was fed back to the FTIR for the determination of photocurrents. The responsivity of the GeSn MSM PDs was then obtained by calibrating the optical responses using a commercial extended InGaAs PD (Thorlabs DET10D2, Thorlabs, Inc., Newton, NJ, USA) to determine the optical responsivity.

## 3. Results and Discussion

### 3.1. Material Characterization

**Figure 2:**
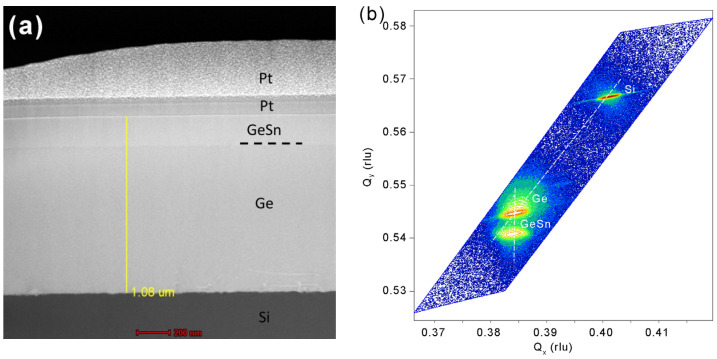
(**a**) Cross-sectional transmission electron microscopy (XTEM) image of the grown GeSn/Ge/Si sample. (**b**) (224) X-ray diffraction reciprocal space mapping (XRDRSM) of the GeSn/Ge/Si sample, revealing a pseudomorphic heterostructure.

Figure 2a shows a cross-sectional XTEM of the grown GeSn sample. Most defects were confined in the region near the Ge/Si interface, and no obvious defects were found near the surface of the Ge VS. The single-crystalline GeSn had a thickness of ~180 nm which was below the critical thickness for plastic relaxation, resulting in a high-quality GeSn layer. As a result, no obvious threading defects were observed in the TEM image of the GeSn layer, as shown in Figure 2a, suggesting the GeSn layer was pseudomorphic to the underlying Ge VS. The threading dislocation density (TDD) of the GeSn layer was estimated to be ~4.7 × 10^7^ cm^−2^ by etch pit density (EPD) methods. Figure 2b shows a (224) XRDRSM of the grown GeSn sample, from which three peaks were observed, associated with the Si substrate, Ge VS, and GeSn layer. From the peak positions, the concentration and strain could be extracted. In addition, the Ge and GeSn peaks were aligned at the same *Q*_x_, suggesting that GeSn was pseudomorphically grown on Ge VS. The diagonal line through the Si peak indicates that Ge VS was almost fully relaxed (~0.1% tensile strain due to the annealing process). The Sn concentration and compressive strain of the GeSn layer were determined to be 4% and 0.57%, respectively.

**Figure 3:**
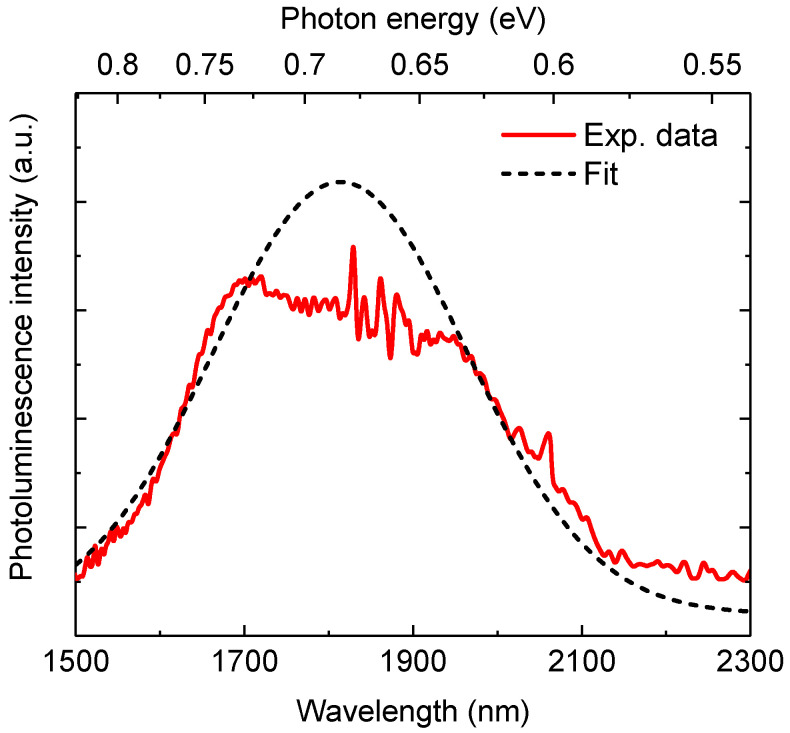
Room-temperature photoluminescence spectrum of the grown GeSn/Ge/Si sample. The signal distribution in the range of 1700–1900 nm is attributed to the atmospheric absorption.

To confirm the bandgap of the grown GeSn sample, photoluminescence (PL) experiments were performed at room temperature using a 532 nm laser as the light source. The emitted PL signals from the GeSn sample were recorded uisng Fourier Transform infrared spectroscopy (FTIR) with ab LN2-cooled InSb photodetector. Figure 3 shows the measured room-temperature PL spectrum from the grown GeSn sample. Although there was signal distortion in the range of 1700–1900 nm due to the atmospheric absorption, a single emission peak was observed near 1800 nm. In addition, the emission peak was asymmetrical, confirming direct-bandgap light emission. The measured PL spectrum was modeled using the modified Gaussian function to obtain the emission peak position; the results are depicted in Figure 3. From the results, the emission peak was determined to be 1810 nm. The emission peak ($E_{\max}$) was related to the direct bandgap energy $E_{g}^{\Gamma }$ via

(1) $$E_{\max}=E_{g}^{\Gamma }+\frac{kT}{2}$$

where *k* is the Boltzmann constant and *T* is temperature. Using the $E_{\max}$ obtained from the room-temperature PL spectrum, we obtained $E_{\max}=0.672\;\mathrm{eV}$. This value was obviously smaller than that of pure Ge (0.8 eV), showing the reduced direct bandgap of the GeSn materials due to Sn-alloying. 

### 3.2. Dark Current, Photogenerated Current, and Gains

**Figure 4:**
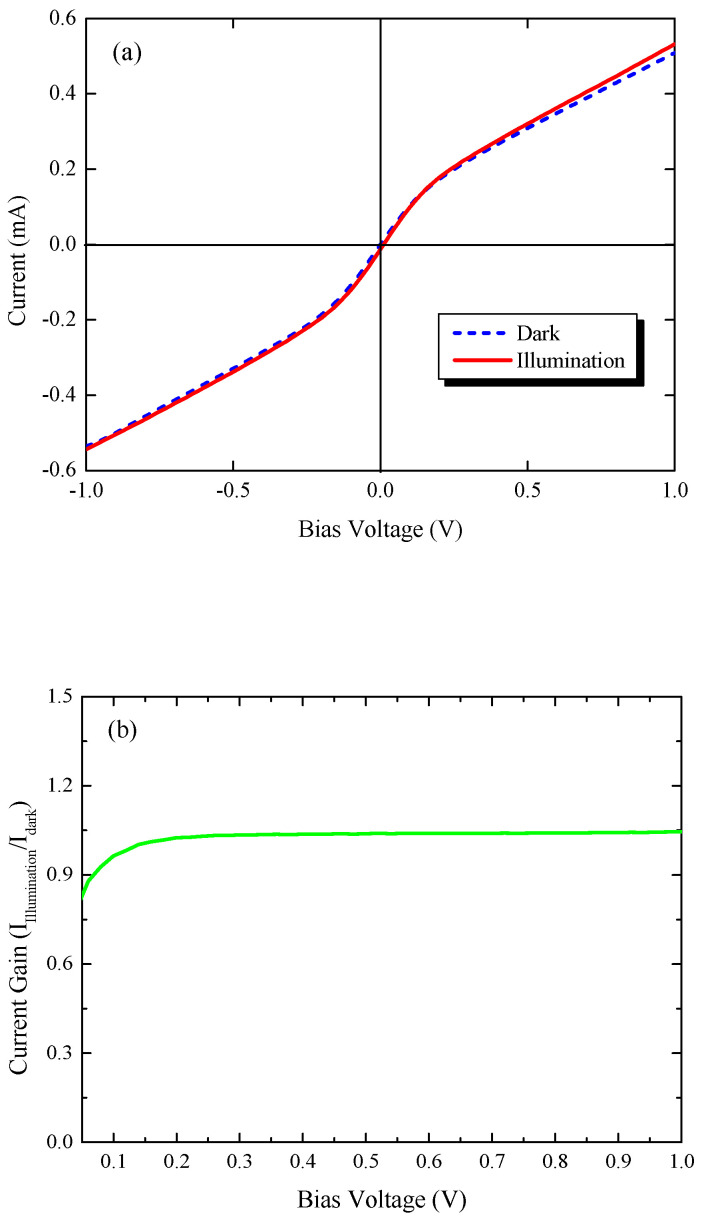
(**a**) Measured dark current and photocurrent variation with bias voltage at *T* = 300 K. (**b**) Measured current gain (I_Illumination_/I_dark_) variation with bias voltage at *T* = 300 K.

Figure 4a shows the current-voltage (I-V) characteristics under dark and illumination conditions. The symmetric nature of the dark current at forward and reverse bias was observed due to the design of the symmetrical electrodes. In addition, the I-V curves were also seen to be nonlinear, this indicated the presence of the Schottky contact between the metal and GeSn layer. The increase in bias voltage increased the band to band tunneling mechanisms; therefore, the dark current increased. In addition, our fabricated GeSn-based MSM PD exhibited a lower measured value of dark current compared to the fabricated Ge-based MSM PD [44], suggesting good material quality. Under illumination with a 1510 nm laser source and an optical power of 7.7 mW, the enhancement of the current confirmed the photodetection ability of the fabricated device. Figure 4b exhibits the current gain which could be obtained from the ratio of current under illumination (*I*_Illumination_) and dark current (*I*_dark_) as a function of bias voltage. The current gain showed a constant nature with applied bias due to the constant enhancement of the current under dark and illumination. 

### 3.3. Spectral Responsivity 

**Figure 5:**
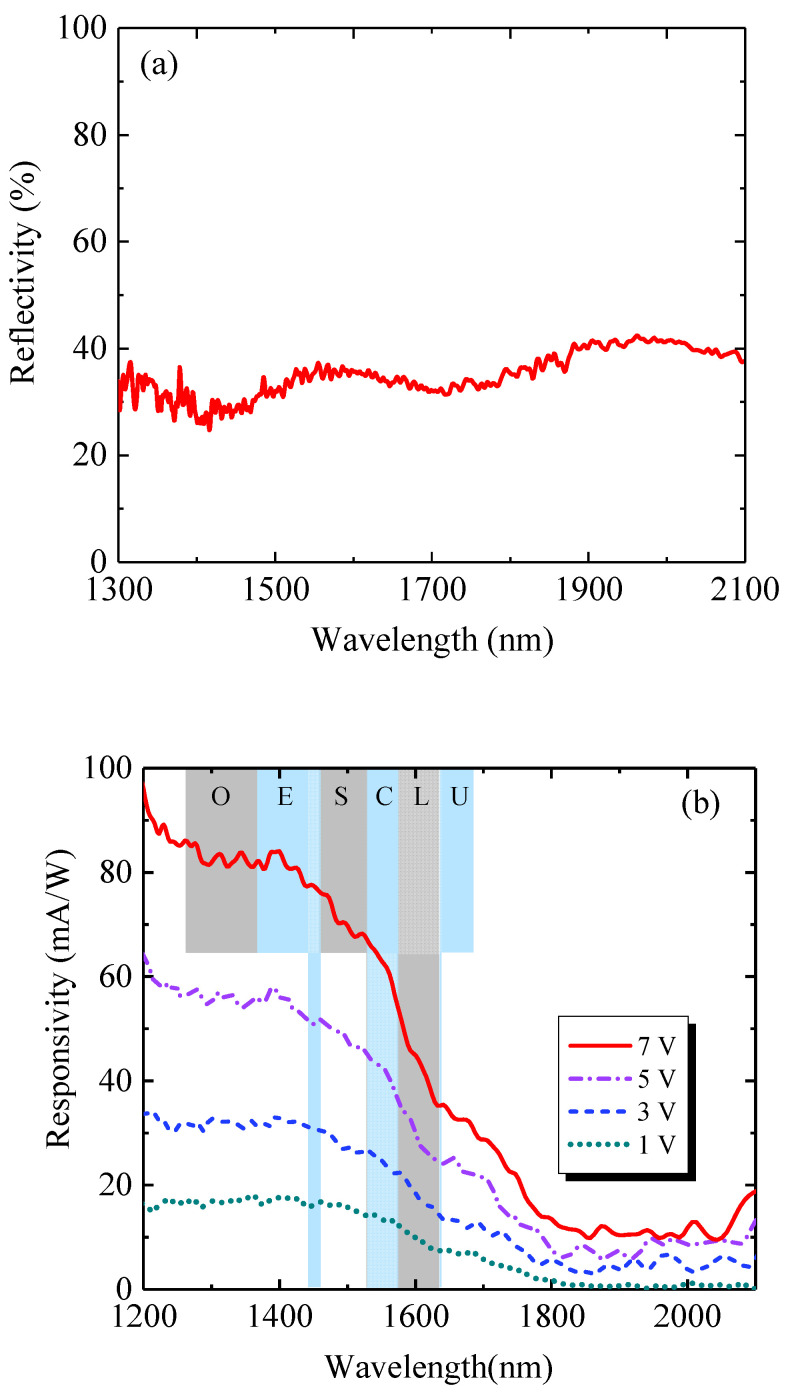
(**a**) Measured reflectivity spectra for the GeSn MSM PDs. (**b**) Measured responsivity spectra of the GeSn MSM PDs with different bias voltages at T = 300 K.

To obtain the maximum spectral responsivity, a SiO_2_ AR layer was employed to reduce the reflection of incident light for MSM PDs. Figure 5a shows the measured reflectivity for the GeSn MSM PDs. For the GeSn MSM PDs, reflectivity showed low values of 20%–30% in the wavelength of 1400 to 2000 nm. The ripple-like features of reflectivity spectra were observed due to the interference between the layers. Figure 5b shows the measured responsivity spectra of the GeSn MSM PDs with different bias voltages at room temperature and the reflectivity spectrum. It can be seen that the responsivity of the device decreased with an increase in the wavelength. From the responsivity at a bias voltage of 1V, the cutoff-wavelength was estimated to be 1800 nm. This extended photodetection cutoff wavelength compared to 1550 nm of Ge PDs was caused by the incorporation of Sn in the active layer, indicating that the lowest direct bandgap of the GeSn active layer was $E_{g}^{\Gamma }$= 688 meV, which is in reasonable agreement with the PL results. The measured results suggested that the photodetection range of our devices entirely covered the telecommunication O-, E-, S-, C-, L-, and U- bands; thus, it is useful for telecommunication applications. Beyond 1800 nm, the optical responsivity was low because only inefficient indirect-gap interband absorption contributed to the optical absorption. Furthermore, the spectral responsivity of the device increased with increasing bias voltage. The enhancement ratios for GeSn MSM PDs for 1, 3, 5, and 7 V were about 84.5%, 219.6%, and 369.6%, respectively, compared to the referential PD for 1V at 1550 nm. The increase in responsivity with bias voltage could be explained based on the high electric field, which enhanced the carrier generation rate and the carrier collection efficiency. However, a high bias voltage caused more power dissipation in the device; therefore, the low bias voltage was preferred for the operation of the device. The applied voltage could be significantly reduced by shrinking the size of the GeSn MSM, while a high responsivity could be maintained. The designed device showed the peak responsivity of 40 mA/W at 1550 nm, which is much higher than that of conventional SiGe-based MSM PDs [45,46,47], showing the unique advantages of GeSn MSM PDs for telecommunication applications. The high spectral responsivity of GeSn MSM PD was due to the direct bandgap nature, high absorption coefficient, and high carrier mobility of GeSn alloy in the active region. In addition, it was also observed that the optical responsivity beyond 1800 nm also significantly increased with increasing applied bias voltage. This observation could be attributed to the Joule heating effect that enhanced lattice vibrations (phonons), resulting in enhanced indirect-gap absorption. Further enhancement in optical responsivity is possible by increasing the Sn content to further increase the absorption coefficient and/or optimizing the device structure to increase the carrier collection efficiency to enable more sensitive short-wave infrared (SWIR) photodetection.

### 3.4. Numerical Analysis

The incorporation of Sn into Ge led to the extension of the absorption edge to the higher wavelength. This was because of the reduction in the direct bandgap of the GeSn alloy with Sn alloying. With an increase in Sn concentration, the Γ-conduction band shifted downward mainly due to the negative bandgap of α−Sn and the bowing effect of the direct bandgap. However, the heavy-hole (HH) band light-hole (LH) band in the valence band shifted upwards with an increase in Sn concentration. Therefore, direct-gap interband transitions contributed to the increased absorption spectra with Sn alloying owing to the increased density-of-states (DOS). To obtain a clear visualization of the effect of Sn concentration on the absorption coefficient, we theoretically calculated the strained electronic band structures using the deformation potential theory [42,48]. Then, the direct band absorption coefficient was calculated by using the Fermi’s golden rule with consideration of a Lorentzian lineshape function [25,49],

(2) $$\alpha (\hbar \omega )=\frac{\pi \hbar e^{2}}{n_{r}c\varepsilon_{0}m_{0}^{2}\hbar \omega }\sum_{m}\int \frac{2dk}{{\left(2\pi \right)}^{3}}{\left|\hat{e}\cdot p_{CV}\right|}^{2}\times \frac{\gamma /\left(2\pi \right)}{{\left[E_{C\Gamma }(k)-E_{m}(k)-\hbar \omega \right]}^{2}+(\gamma /2{)}^{2}}$$

where n_r_ is the refractive index; c is the velocity of light in free space; e is the electronic charge; ħ is the reduced Planck’s constant; m_0_ is the rest mass of an electron; ɛ_0_ is the free space permittivity; ω is the angular frequency of incident light; ${\left|\hat{e}\cdot p_{CV}\right|}^{2}$ is the momentum matrix; γ is the full-width-at-half-maximum (FWHM) of the Lorentzian lineshape, whose value 15 meV was used in this study; *E*_CΓ_ (**k**) and *E*_m_(**k**) are the electron and hole energy in the Γ-valley conduction band (CB) and valance band (VB), respectively, which were calculated using a multi-band k·p method by considering the strain effect [33,42]. The summation over m represents all interband transitions from the VB (HH and LH bands) to the direct CB. For the indirect-band absorption, because the probability of the indirect-gap transition was much smaller than that of the direct-gap transition, we neglected the indirect-band absorption effect in this study. The parameters for GeSn alloys could be evaluated from the linear interpolation of the Ge and Sn, as shown in Table 2. The direct bandgap bowing parameter of GeSn alloy was *b*_Γ_ = 2.42 eV [33].

**Figure 6:**
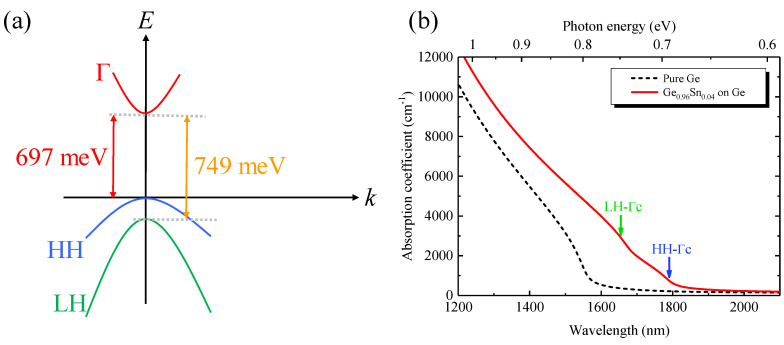
(**a**) Band structure of pseudomorphic GeSn with 4% Sn content on Ge in which heavy-hole (HH) and band light-hole (LH) split due to compressive strain. (**b**) Calculated absorption spectra for pure Ge and GeSn with 4% Sn composition pseudomorphically grown on Ge VS at T = 300 K.

Figure 6a shows a schematic band diagram of pseudomorphic Ge_0.96_Sn_0.04_ on Ge with respect to wavenumber (k)at T = 300 K. The pseudomorphic growth of GeSn with 4% Sn on Ge VS exerted a compressive strain (~0.57%) which split the degeneracy of the HH and the LH bands. It is clearly shown from Figure 6a that the HH band shifted above the LH band, and their separation energy became larger with increasing Sn concentration due to the larger compressive strain. Therefore, two possible direct interband transitions contributed to the optical absorption: the direct transition from the HH band to the Γ-conduction band (HH→Γc) and from the LH band to the Γ-conduction band (LH→Γc). The calculated transition energies for the HH→Γc and LH→Γc transitions were 697 and 749 meV, respectively, which are in good agreement with the experimental results. Figure 6b shows the calculated absorption spectra of pure Ge and pseudomorphic Ge_0.96_Sn_0.04_ on Ge at T = 300 K. The overall absorption coefficient was the superposition of the direct interband transitions: HH→Γc and LH→Γc; therefore, a cusp-like feature was shown in the calculated absorption spectra. The calculated result shows that the absorption coefficient decreased with an increasing wavelength. The calculated result also shows that the optical cutoff wavelength for GeSn alloy shifted towards the longer wavelength, which considerably increased the photodetection range of the proposed device. The calculated result also shows that the absorption coefficient increased with increasing Sn concentration. For Ge_0.96_Sn_0.04_ on Ge, the absorption coefficient increased by ~3.3 times more than pure Ge at 1550 nm. This behavior can be explained based on the redshift in the absorption edges with increasing Sn concentration due to the shrinkage of the bandgap energies from the Sn alloying. Further increases in the Sn content can redshift the direct-gap absorption edge and thus extend the photodetection range of the GeSn MSM PDs for important short-wave infrared applications.

## 4. Conclusions

In conclusion, we have demonstrated a GeSn MSM PD monolithically grown on Ge buffered Si substrates. The GeSn active layer was grown on a silicon substrate with good material quality as the optical absorber, which had a lower bandgap than that of pure Ge, extending the photodetection region. The responsivity experiments show enhanced spectral responsivity and low dark current compared to the existing SiGe-based MSM PDs. Furthermore, the responsivity increases with an increase in bias voltage due to the enhanced electric field. With the extended photodetection range, planar structures and CMOS compatibility, Si-based short-wave infrared GeSn PDs are promising for a wide range of applications.

## Figures and Tables

**Table 1:** Materials and dimensions of the GeSn metal-semiconductor-metal photodetectors (MSM PDs).

Layer	Material	Thickness (nm)
Substrate	Si	150
Virtual Substrate	Ge	900
Active Layer	Ge_0.96_Sn_0.04_	180
Top Passivation Layer	SiO_2_	180

**Table 2:** Parameters for Ge and Sn at T = 300 K.

Parameters	Ge	Sn
Lattice Constant *a* (Å)	5.6573 [19]	6.4892 [19]
Electron Effective Masses		
Electron *m_Γ_/m_0_*	0.038 [19]	0.058 [19]
Heavy Hole *m_HH_/m_0_*	0.28 [34]	
Light Hole *m_LH_/m_0_*	0.044 [34]	
Band gap *E_gΓ_* (eV)	0.7985 [19]	−0.413 [19]
Spin-orbit splitting Energy *Δ_0_* (eV)	0.29 [19]	0.80 [19]
Average Valance Band Energy *E_VaV_* (eV)	0 [19]	0.69 [19]
Luttinger’s parameters		
γ_1_	13.38 [19]	−14.97 [19]
γ_2_	4.24 [19]	−10.61 [19]
γ_3_	5.69 [19]	−8.52 [19]
Deformation Potential		
*a_c_* (eV)	−8.24 [19]	−6.00 [19]
*a_v_* (eV)	1.24 [19]	1.58 [19]
*b_v_* (eV)	−2.90 [19]	−2.70 [19]
Elastic Constants		
*C_11_* (GPa)	128.53 [19]	69.00 [19]
*C_12_* (GPa)	48.26 [19]	29.30 [19]
*C_44_* (GPa)	68.30 [19]	36.20 [19]
Electron mobility *µ_n_* (cm^2^/V-sec)	3900 [28]	2940 [28]
Hole mobility *µ_p_* (cm^2^/V-sec)	1900 [28]	2990 [28]
Optical Energy *E_p_* (eV)	26.3 [19]	24.0 [19]
Refractive Index *n_r_*	4.051 [28]	5.791 [28]
Dielectric Constant *ε_r_*	16.2 [28]	24.0 [28]
